# Relationship between the obstacle height cognition and step movement in the elderly

**DOI:** 10.1186/1880-6805-31-27

**Published:** 2012-10-16

**Authors:** Sohee Shin, Shinichi Demura, Tsuneo Watanabe, Haruka Kawabata, Hiroki Sugiura, Toshio Matsuoka

**Affiliations:** 1 Department of Sports Medicine and Sports Science, Gifu University Graduate School of Medicine, 1-1 Yanagido, Gifu City, 501-1194, Japan; 2 Kanazawa University Graduate School of Natural Science & Technology, Kakuma, Kanazawa, Ishikawa, 920-1192, Japan

**Keywords:** Step over, Tripping, Fall

## Abstract

**Background:**

This study examines the effect of obstacle height cognition (OHC) on single-leg forward step (SFS) and Obstacle-SFS.

**Methods:**

In the SFS test, participants stepped 25 cm forward with one leg and returned it to its original position five times as quickly as possible. The Obstacle-SFS added an obstacle to the above condition in the SFS test. The participants were divided into two groups: tripping group, which tripped over an obstacle in the Obstacle-SFS test; and non-tripping group, which did not trip. Parameters were step time (T), the time it took to step forward (F), and the time it took to return to the original position (R). The OHC was determined by the difference between the elevated leg’s height and the obstacle height (10 cm), which was set at 60 cm in front of the participant.

**Results:**

OHC showed a significant and moderate relationship with all parameters of Obstacle-SFS (OSFS-T, OSFS-F and OSFS-R). The tripping group had significantly larger values in the OHC, OSFS-T and OSFS-F than the non-tripping group.

**Conclusions:**

In conclusion, the differences in obstacle height cognition ability may affect Obstacle-SFS movement.

## Background

For older persons, physical fitness declines with age. However, it is unclear whether the older persons themselves grasp the decline of their physical fitness adequately. People judge their physical fitness in conscious and unconscious ways. When they cannot correctly assess their condition, problems ensue [1].

When sensing an impending fall, people step forward quickly and avoid it by returning to a stable base of support. Shin and Demura [2-4] have evaluated older persons’ fall-related physical fitness, noted the movement involved in making a new base of support by stepping forward, and developed the single-leg forward step test (SFS test). They have also evaluated the usefulness of the test. In the SFS test, participants must step forward and return the leg to its original position quickly. The supporting and stepping legs require considerable leg strength and balance ability, because braking power is needed to control the body’s forward momentum. It was reported that the test using this movement has a close relationship to the fall risk score and is useful for evaluating the fall-related physical fitness of older persons [5]. Shin *et al*. [6] reported that Obstacle-SFS reflects performance characteristics of older people who have experienced a fall by tripping, and is thus useful for evaluating the characteristic movements of older people who are prone to trip.

From the results of a previous study [5], older people who are prone to trip tend to be inferior in Obstacle-SFS movement. Hence, this test may be able to predict how older people might trip in the future. Tripping over an obstacle is one of the biggest causes of falling in older people [7-9]. According to Robinovitch *et al*., [10] older people tend to overestimate their own physical fitness, and they suggested that older people must adequately evaluate physical fitness and behave accordingly, in order to avoid trips and falls. Older people need to assess an obstacle’s height properly to avoid a fall during a step. Tripping over an obstacle because one is physically unable to raise a leg is a concern, but fall risk also increases because of unstable or excessive lifting of the leg. To date, several studies have been performed to elucidate the relation between the Obstacle-SFS movement and leg strength, balance ability, and visual recognition ability [5]. However, the relationship between Obstacle-SFS and the cognition of the height of an obstacle has not been well examined.

This study hypothesized that older people with inferior obstacle height cognition (OHC) also have inferior Obstacle-SFS performances. The results of this study have provided important data regarding tripping and they will help to develop a preventive method against tripping and falling in older people.

This study examined the effect of OHC on the single-leg forward step (SFS) and Obstacle-SFS.

## Methods

### Participants

The participants were 43 healthy older women who can walk independently (age 77.0 ± 5.30 years, height 148.0 ± 6.29 cm, weight 51.9 ± 16.8 kg). The age of the participants ranged from 68 to 91 years. Among the participants, 27 exercised with low-to-moderate intensity one time per week. When asked about their physical fitness and health, 32 (74.4%) persons answered ‘normal’, and 31 (72.1%) answered ‘excellent’. It is assumed that the older people in this study have high physical fitness and are in good health. Shin *et al*., reported that the Obstacle-SFS test may be useful to assess whether the characteristics of geriatric tripping and OHC influence Obstacle-SFS performance. It is assumed that movement characteristics when stepping over an obstacle varied between individuals who tripped and who did not. Hence, this study divided participants into two groups: the group of individuals (tripping group, N = 12) who tripped on an obstacle in the Obstacle-SFS test and the group of individuals (non-tripping group, N = 31) who did not trip, and then compared the groups with respect to Obstacle-SFS movement and OHC characteristics. The purpose and procedures of this study were explained in detail, and informed consent was obtained from all participants. This study was approved by the Kanazawa University Department of Education Ethical Review Board.

### Single leg forward step test (SFS test)

**Figure 1:**
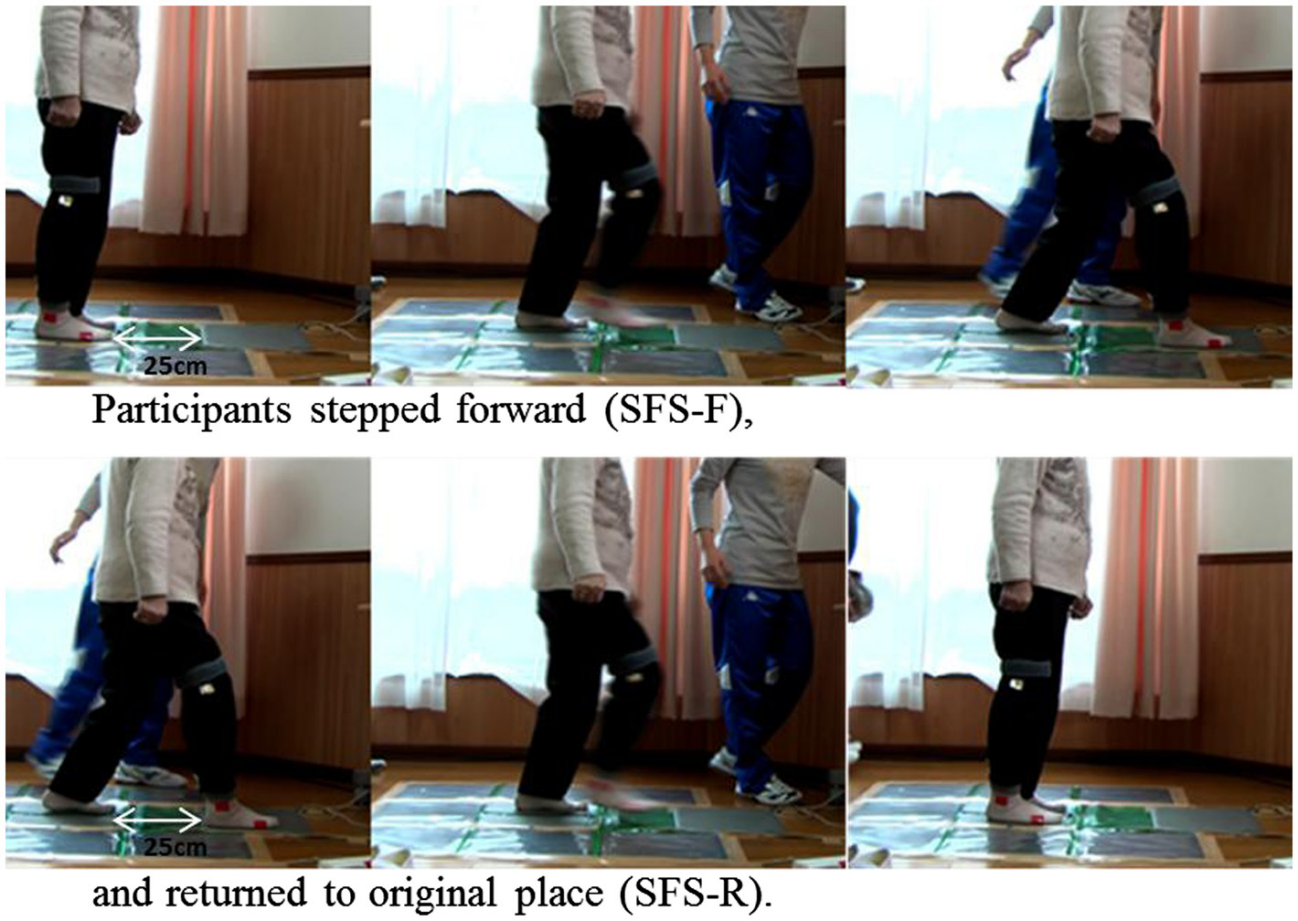
**Single-leg Forward Step test (SFS) test.** Note: Participants repeated the above measurement five times, as fast as possible.

Participants stood barefoot on a step sheet, arms relaxed, in a quiet room. They were asked to look at the line drawn 25 cm in front of them while stepping forward over the line (Figure 1). From the results of the pilot experiment, it was determined that the physical burden on older participants increases when they are asked to perform twice on both legs, and that for some participants, one leg is preferred over the other as a support leg. Hence, before the measurement, participants were asked which leg was easier to stand on or to operate, by Demura’s assessment [11]. They used the preferred leg as the supporting leg, stepped forward with the other leg, and returned to an original position five times as quickly as possible. 

The measurement was conducted once after one practice trial, but was repeated if they did not step forward over the line. The step test was performed using the step sheet (Takei Inc., Niigata, Japan) which can measure swing time from the lifting of one leg to landing, based on foot pressure information.

### Obstacle-single leg forward step test (Obstacle-SFS test)

**Figure 2:**
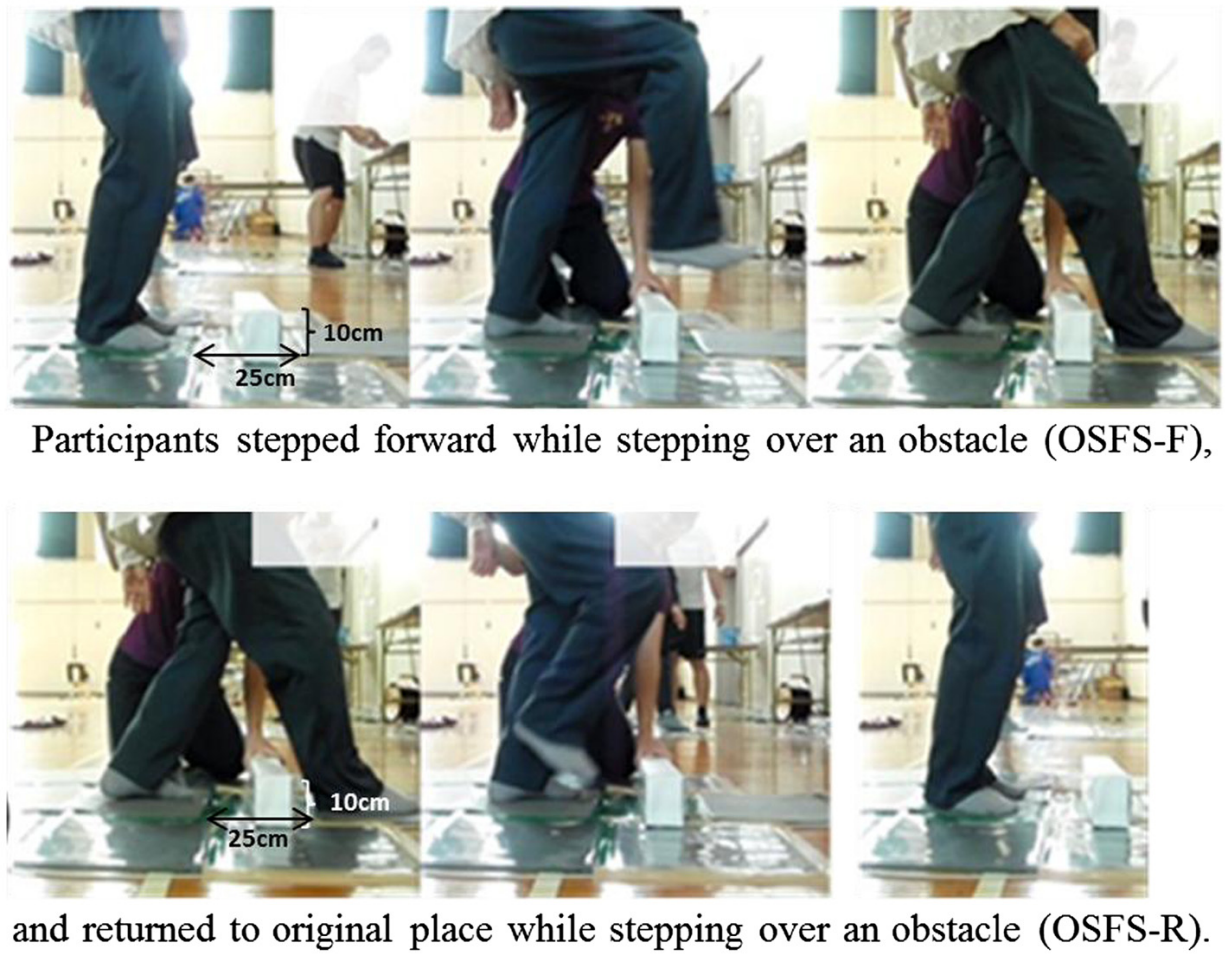
**Obstacle Single-leg Forward Step test (Obstacle-SFS) test.** Note: Participants repeated the above measurement five times, as fast as possible.

The Obstacle-SFS test is one that sets an obstacle in an SFS test and is measured with the same method as the SFS test (Figure 2). Participants stood barefoot on a step sheet with relaxed arms in a quiet room and were asked to look at the obstacle. They stood with the supporting leg, stepped forward over the obstacle with another leg, and returned to an original position five times as quickly as possible. The step length from the start spot was 25 cm and the obstacle was set at the midway point. A tester held the obstacle lightly in place so that it did not move if hit by the participant during the task. The measurement was conducted once after one practice trial, but was repeated if they tripped over the obstacle. A mean time of five steps when participants did not trip during the trial was taken into account for analysis. Tripping and non-tripping groups were classified on the basis of the results of the first trial. When an individual from the tripping group tripped, the data were not used for analysis and the individual was made to perform the task again and the data from a trial when the individual did not trip were used. The step test was performed using the same step sheet (Takei Inc.) as the SFS test.

### Evaluation parameters

A step was divided into two phases: first, the leg is raised off a step sheet from the start point and is placed on the forward step sheet, and in the second phase the leg is raised off the latter sheet and is returned to the original position. Forward phase time and returning phase time were a mean of five total times for the two phases, respectively.

1. Step time (T; Total time, Seconds).

The time accounted for one step is from the moment the leg is raised off the step sheet at the start spot to the point when the leg is returned back to the original position. A mean of five step times was used as a parameter for comparison.

2. Forward phase time (F, Unit: Seconds).

The forward phase was considered to be the time from the moment the leg is raised off the step sheet at the start spot until it is placed on the front step sheet. A mean of five total forward phase times was used as a parameter for comparison.

3. Returning phase time (R, Unit: Seconds).

The returning phase time was considered to be the time from the moment the leg is raised off the forward step sheet until it is placed back on the original step sheet. A mean five total returning phase times was used as a parameter for comparison.

FS-T, SFS-F and SFS-R are abbreviations of the above parameters in the SFS test, and OSFS-T, OSFS-F and OSFS-R are those of the above parameters in the OSFS test. The mean and SD of the above parameters were calculated. Data over the mean ± 3SD were judged as the outliers and the data after eliminating these values were used as statistical analyses.

### Obstacle height cognition (OHC, unit: cm)

**Figure 3:**
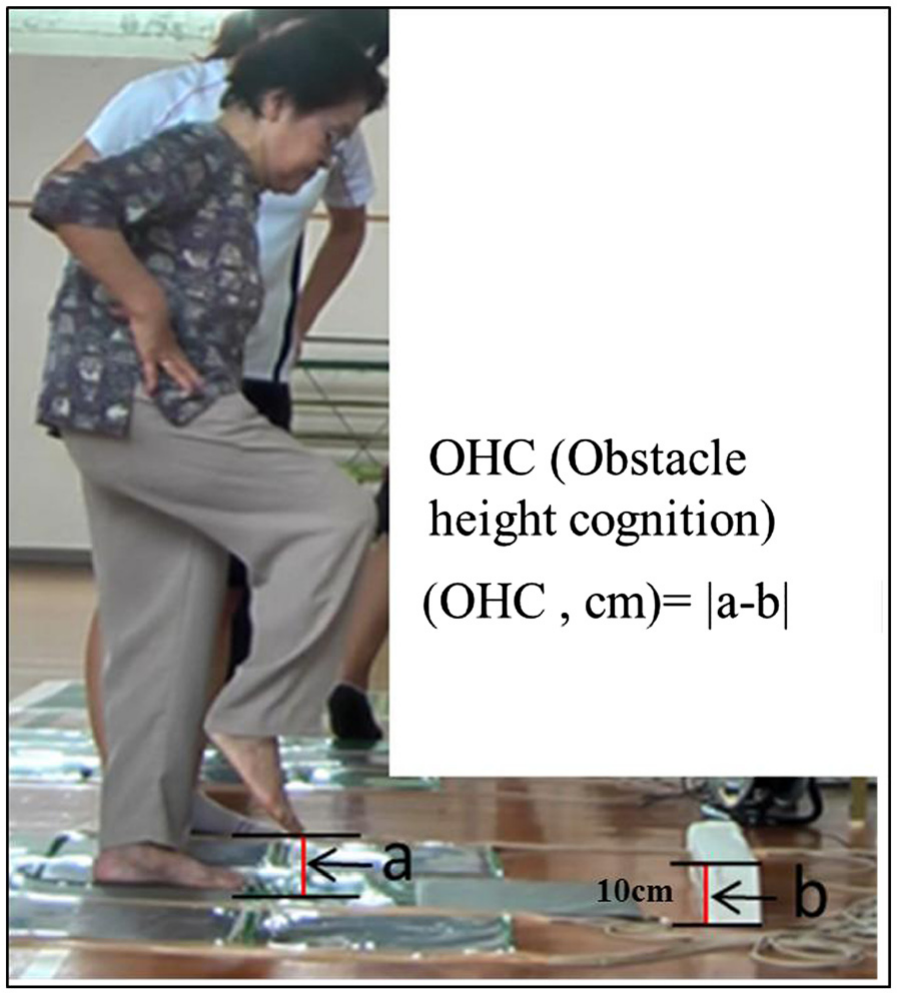
**The obstacle height cognition.** OHC was calculated as the difference between the real raised leg height (**a**) and the obstacle height (**b**) (The absolute value of a-b in Figure 3).

OHC was measured using a video camera (Panasonic HC-X900M, Tokyo Japan). The camera was set at a distance of 2.6 m from the step sheet, and the data obtained were captured as still images. Participants were asked to lift one leg to the height of the 10 cm tall obstacle after viewing it. The obstacle was set up at 60 cm in front of the supporting leg. OHC was measured two times after one practice trial per leg. OHC was calculated as the difference between the real raised leg height (a) and the obstacle height (b) (the absolute value of a-b in Figure 3). The smallest OHC from the trials is used for statistical analysis.

### Statistical analyses

To examine mean differences between tripping and non-tripping groups in the Obstacle-SFS test for the OHC and step tests, an unpaired *t* test was used. An effect size was calculated to examine the size of the mean differences. Pearson’s correlation was used to examine relationships between the OHC and SFS and Obstacle-SFS tests. The probability level of *P* <.05 was indicative of statistical significance.

## Results

### The group differences between the SFS and obstacle-SFS

Table 1 shows the differences between tripping and non-tripping groups for each parameter. The tripping group had significantly larger results in the obstacle height cognition (OHC), OSFS-T and OSFS-F than non-tripping group, and the effect size of the OHC was very large (ES: 1.26).

**Table 1:** The group differences between the SFS and Obstacle-SFS

	**Group**	**Mean**	**SD**	**t-value**	**ES**
OHC(Obstacle height cognition)	Tripping	4.99	3.76	3.48	1.26
	non-Tripping	1.77	1.84		
SFS-T	Tripping	0.52	0.08	0.39	0.13
	non-Tripping	0.51	0.11		
SFS-F	Tripping	0.52	0.08	0.92	0.31
	non-Tripping	0.49	0.10		
SFS-R	Tripping	0.53	0.11	0.04	0.01
	non-Tripping	0.52	0.14		
OSFS-T	Tripping	0.70	0.14	2.07	0.70
	non-Tripping	0.61	0.13		
OSFS-F	Tripping	0.71	0.15	2.67	0.91
	non-Tripping	0.59	0.13		
OSFS-R	Tripping	0.69	0.15	1.38	0.47
	non-Tripping	0.63	0.14		

*: *P* <0.05; *F* forward phase time, *OSFS*, obstacle *SFS* Single leg forward step, *R* returning phase time, *SD*, standard deviation, *SFS* single leg forward step, *T* the time of steps. The tripping group had significantly larger results in the OHC, OSFS-T and OSFS-F than the non-tripping group, and the effect size of the OHC was very large (ES: 1.26).

### The relationship between OHC and each step test

Table 2 shows the correlations between the OHC and each step test. The OHC had significant and moderate values with all parameters (OSFS-T, OSFS-F and OSFS-R) of the Obstacle-SFS test, but not with all parameters of the SFS test.

**Table 2:** The relationship between OHC and each step test

	**OHC**	**SFS-T**	**SFS-F**	**SFS-R**	**OSFS-T**	**OSFS-F**	**OSFS-R**
OHC							
SFS-T	0.32						
SFS-F	0.31	0.92*					
SFS-R	0.29	0.96*	0.78*				
OSFS-T	0.49*	0.77*	0.74*	0.71*			
OSFS-F	0.47*	0.62*	0.65*	0.55*	0.92*		
OSFS-R	0.44*	0.80*	0.74*	0.76*	0.94*	0.73*	

*: *P* <0.05; *F*, forward phase time, *OHC* obstacle height cognition (unit: cm); *OSFS* Obstacle-SFS; *R* returning phase time, *SFS* Single leg forward step, *T* the time of steps.

## Discussion

This study aimed to examine the effect of OHC on the SFS and Obstacle-SFS tests. To evaluate older persons with lower physical fitness, safe tests should be selected. In addition, it is preferable that the test content relates closely to their daily life activities and is available for rehabilitation and functional recovery [12].

This study used the step test, during which older persons shift their center of gravity forward and backward. This test is useful because it can be performed in a small space, and it is a simple and safe movement that older participants can easily understand. In addition, Shin *et al*. [2] reported that the step test has high reliability (intraclass correlation coefficient = 0.90). The step test was designed by considering the following points: 1. when almost falling, older people step forward onto one leg to keep their base of support and to prevent the fall; 2. a decrease in leg strength and balance ability as well as the range of motion of the hip, knee and ankle joints are associated with factors related to the fall; and, 3. a screening test should use movements that older persons can easily understand. With the consideration of these above points, the Obstacle-SFS examined the movement characteristics of older persons who trip easily by adding an obstacle to the SFS test.

The OHC of the older persons who tripped during the Obstacle-SFS movement was large. Shin *et al*. [6] divided participants into a group with fall experience and a group without it, and compared Obstacle-SFS test performances. The fallers by trip scored significantly higher in all parameters of the Obstacle-SFS test than the non-fallers. Hence, it was judged to be a useful test for evaluating the movement characteristics of older persons who trip easily. The study presented here clarified that older people with inferior Obstacle-SFS movement have a large OHC. The results presented here may support the hypothesis of the Shin *et al*. [6] study and the results of previous studies. There may be two patterns by which older persons trip over an obstacle: tripping over an obstacle due to not being able to recognize the obstacle adequately, and tripping because they could not lift a leg in spite of recognizing the height of an obstacle (overestimating their own physical fitness). Both situations are commonly experienced by community-dwelling older persons.

There were non-significant relationships between the OHC and the SFS test without an obstacle. Although it is difficult to distinguish both tests by only the OHC, it is assumed that the Obstacle-SFS test can also evaluate the coordination ability required to visually recognize an obstacle and to lift the leg up to the obstacle height, in addition to the leg strength and dynamic balance required in an SFS test. In short, the step-over movement may be affected largely by dissonance in cognition and physical behavior in the older people who trip easily. Suzuki *et al*. [1] reported that older people tend to over-estimate their own physical fitness. If older people with inferior physical fitness over-estimate their abilities, they cannot respond adequately to the external environment and could easily break their posture balance. In short, it becomes the cause of tripping or staggering by hitting an obstacle. This is supported by the present results.

On the other hand, since all phases (OSFS-T, OSFS-F and OSFS-R) in the Obstacle-SFS test showed significant relationships with OHC, it is inferred that all phases are affected by OHC; the OSRS-R phase showed an insignificant difference between tripping and non-tripping groups. In the case of the OSFS-R phase, the older persons step over an obstacle, depending on the sense of a lifted leg without visual information because the obstacle is located behind the stepped leg. Essentially, this phase is different from the OSFS-F phase, during which they can step over an obstacle while seeing it. Hence, it is inferred that the older persons who could not lift their leg adequately tripped over the obstacle. The OHC of this study was measured from data obtained using a video camera after controlling for the position of the participants, obstacle, and camera. However, the positioning of the subject’s vision could not be controlled completely. In addition, the effect of balance ability and leg strength on the Obstacle-SFS movement was not examined.

Now it is necessary to focus on balance ability, leg strength and visual information, and to examine the effect of these factors on tripping in older people and the relationship with the OHC.

## Conclusions

In conclusion, the older persons who tripped over the obstacle during an Obstacle-SFS test have a larger OHC than those who did not trip. The OHC relates to all parameters of an Obstacle-SFS test and the differences in ability in OHC affects Obstacle-SFS test performances.

## Abbreviations

OHC: obstacle height cognition; SFS: single-leg forward step.

## Competing interests

The authors declare that they have no competing interests.

## Authors’ contributions

SS and SD were a general coordinator and did the study design. TW, HK, HS and TM were involved in data collection, data interpretation, and result analysis and literature search. All authors read and approved the final manuscript.
